# Protective effect of apolipoprotein E epsilon 3 on sporadic Alzheimer’s disease in the Chinese population: a meta-analysis

**DOI:** 10.1038/s41598-022-18033-x

**Published:** 2022-08-10

**Authors:** Qian Chen, Ting Wang, Deying Kang, Lei Chen

**Affiliations:** 1 Department of Clinical Research Management, Center of Biostatistics, Design, Measurement and Evaluation, West China Hospital of Sichuan University, Chengdu, Sichuan China; 2 Department of Evidence-Based Medicine and Clinical Epidemiology, West China Hospital, Sichuan University, Chengdu, Sichuan China; 3 Department of Clinical Research Management, West China Hospital, Sichuan University, No. 37, Guoxue Alley, Chengdu, 610041 Sichuan China; 4 Department of Neurology, West China Hospital, Sichuan University, Chengdu, Sichuan China

**Keywords:** Alzheimer's disease, Risk factors

## Abstract

Alzheimer’s disease (AD) is fast becoming one of the most expensive, deadly and burdensome diseases in this century. It has the fastest-growing disease burden in China. Apolipoprotein E (APOE) polymorphic alleles are generally considered to be the primary genetic determinant of AD risk: individuals with the E4 allele are at increased risk of AD compared with individuals with the more common E3 allele. Since the intensity of the association varies among different ethnic groups, a separate meta-analysis of the Chinese population is needed. We searched Chinese and English databases to sift through literature over the past 20 years. Data on the APOE genotype and AD were collected for correlation analysis. OR was calculated according to APOE allele and genotype. A publication bias analysis and sensitivity analysis were performed, and the main results were further verified by subgroup analysis. The 116 eligible studies enrolled 23,396 patients with AD and 25,568 healthy controls. The study subjects covered at least 30 of the 34 provincial-level administrative regions (including Taiwan). The partial sex ratio was as follows: AD male/female; 10,291/11,240; control male/female, 11,304/12,428, ${\upchi }^{2}$ = 0.122, *P* = 0.727. The results of the meta-analysis of alleles showed that I^2^ > 50% and Q statistics were significant for all genotypes; therefore, the random effect model was selected. The frequency of the ApoE ε4 allele in AD was higher than that in healthy controls, and the difference was statistically significant (OR 2.847, 95% CI [2.611–3.101], *P* < 0.001). The frequencies of ApoE ε3 and ε2 in AD were lower than those in healthy controls, and the differences were statistically significant (ε3: OR 0.539, 95% CI [0.504–0.576], *P* < 0.001; ε2: OR 0.771, 95% CI [0.705–0.843], *P* < 0.001). The results of the meta-analysis of AD genotype showed that ApoE ε2/ε4 (OR 1.521, 95% CI [1.270–1.823], *P* < 0.001), ε3/ε4 (OR 2.491, 95% CI [2.267–2.738], *P* < 0.001) and ε4/ε4 (OR 5.481, 95% CI [4.801–6.257], *P* < 0.001) allele genotype frequencies were higher than those of the healthy controls. The differences were all statistically significant. Moreover, the ApoE ε2/ε2 (OR 0.612, 95% CI [0.504–0.743], *P* < 0.001), ε2/ε3 (OR 0.649, 95% CI [0.585–0.714], *P* < 0.001) and ε3/ε3 (OR 0.508, 95% CI [0.468–0.551], *P* < 0.001) genotypes were less frequent in patients with AD than in healthy controls, and the differences were statistically significant. The results of the sensitivity analysis and subgroup analysis were consistent with those of the whole model. These results provide support for the protective effect of the ApoE ε3/ε3 genotype against the development of AD. This research is the most comprehensive meta-analysis of the correlation between APOE and AD in the Chinese population by analysing the distribution of the APOE gene in patients with AD reported in the last 20 years. It was concluded that the APOE ε3 allele had a protective effect against sporadic AD in the Chinese population, with great significance, and that its protective effect was stronger than that of the ε2 allele.

## Introduction

Alzheimer’s disease (AD) and other dementias are fast becoming one of the most expensive, lethal and burdening diseases of this century^1,2^. China had more cases of AD in 2020 than any other country in the world^3^. The 2017 China Burden of Disease Study, which was published in The Lancet, reported that AD and other dementias were 8th in the Years of Life Lost YLL) ranking. It ranked 28th in 1990, and this is the fastest-growing disease among the top 10. Additionally, the YLLs to AD in each province are different^4^: therefore, data covering the whole country must be studied. The burden of dementia seems to be increasing faster than is generally assumed by the international health community^5^.

The risk of AD is 60–80% dependent on heritable factors, with more than 40 AD-associated genetic risk loci already identified, among which apolipoprotein E (APOE) alleles have the strongest association with the disease^1^. The mechanism via which the ApoE ε4 protein leads to increased Aβ deposition has been difficult to pinpoint^6^. No evidence has emerged that Aβ production is significantly elevated in cells that coexpress APP with the ApoE ε4 protein vs. the ApoE ε2 or ApoE ε3 proteins^7^. Three common polymorphisms in the *ApoE* gene, ε2, ε3 and ε4, including six genotypes (three homozygous (ε2/ε2, ε3/ε3 and ε4/ε4) and three heterozygous (ε3/ε2, ε4/ε2 and ε4/ε3) genotypes)^8^, result in a single amino acid change in the ApoE protein. Of these alleles, ε3 is the most common, followed by ε4 and ε2, although these frequencies vary between populations^9,10^. ApoE polymorphic alleles are the main genetic determinants of AD risk: individuals carrying the ε4 allele are at increased risk of AD compared with those carrying the more common ε3 allele^11^. Conversely, the ε2 allele has a putative protective effect that is associated with longevity and a lower AD risk^12^.

Although numerous studies have demonstrated this association, inconsistency was presented regarding its degree in different ethnic and geographical groups^13^. Previously, a small-sample meta-analysis of the Chinese population also proposed a different point of view^10^. Therefore, association analysis between APOE gene polymorphisms and AD in Chinese people should be conducted independently rather than directly referring to international reports. The purpose of conducting this meta-analysis was to reduce heterogeneity and summarise the published evidence on the prevalence of the ApoE polymorphism among patients diagnosed with AD in the Chinese population.

## Methods

Between 1 March 2021 and 30 April 2021, we searched PubMed, Embase, Central, China National Knowledge Infrastructure, Wan-fang and VIP databases for articles published from 2000 to 2020. We used the search term ‘Alzheimer’s disease’ in combination with the following terms: ‘China’, ‘Chinese’, ‘APOE’ and ‘apolipoprotein E’. The equivalent Chinese terms were used in the Chinese databases. We also searched the reference lists of the articles identified using this search strategy and selected those that were judged to be relevant. The publication types included peer-reviewed journal publications and postgraduate theses.

The selection criteria were as follows:

1. case–control study;
2. Chinese population (living);
3. any patient group with AD (without complications) or a certain type of AD;
4. the control group included healthy people;
5. detailed APOE genotypes were reported in the case group and the control group, and all subjects were clearly genotyped;
6. there were clear clinical diagnostic criteria.

The exclusion criteria (exclusion after reading the full text) were as follows:

1. Obvious logical errors in the data (the results were inconsistent, and the correct data could not be obtained according to other information. The error included in, but not limited to, the table data was inconsistent with text descriptions).
2. The research objectives included treatment and diagnosis.

The exclusion criteria (exclusion after the completion of information collection) were as follows:

1. Duplicate reporting of the same study (based on identical sample size, baseline, the results and overlapping authors).
2. The scoring criteria for this study were formulated according to *The Newcastle–Ottawa Scale* (CODING MANUAL FOR Case–Control STUDIES)^14^. The included literature was evaluated, and articles with scores < 6 were eliminated.

### PubMed search strategy

#1 apolipoprotein E/ or APOE.mp.

#2 limit 1 to yr = ‘2000–2020’.

#3 AD.mp.

#4 Alzheimer’s disease/or Alzheimer.mp.

#5 China.mp. or China/.

#6 Chinese/ or Chinese.mp.

#7 #3 or #4.

#8 #5 or #6.

#9 #1 and #2 and #7 and #8.

#10 limit #9 to the English language.

Last search date: 2021-04-30.

The following information was recorded in the file: database name, publication title, authors, corresponding author affiliation, published date, journal name, study area (hospital or community), province, AD diagnostic criteria, age of onset, subgroup analysis or not, control group matched or baseline comparable, AD subtype (sporadic AD or familial AD), age of the AD and control groups, sex of AD and control groups, sample size of the AD and control groups and ApoE allele and genotype of the AD and control groups.

### Quality assessment and data extraction

Two investigators independently extracted the data. Any disagreement was resolved by discussion with a third expert. A consensus was reached on all of the items. The data retrieved from the reports included the first author, publication year, age distribution, sex ratio, number of cases and controls, distribution of genotypes and the diagnosis criteria of AD. When authors did not report the absolute or percent ApoE allele frequency, we estimated it from the reported ApoE genotypes.

### Outcomes

A risk factor analysis of the apolipoprotein E genotype in AD was performed in the Chinese population. The primary outcomes included the prevalence of APOE ε3/ε3 in the AD and control groups and the odds ratios (ORs) (and 95% CI) for AD across APOE ε3/ε3. The secondary outcomes included the prevalence of APOE genotypes and alleles in the AD and control groups and the ORs (and 95% CI) for AD across APOE genotypes and alleles.

### Statistical analysis

Data from each ascertainment case–control study group were then pooled together. Variations in the sex of the participants in the study groups were compared using the *χ*^2^ test of homogeneity to exclude correspondingly heterogeneous data sets. Meta-analyses of the case–control study groups were conducted using the Mantel–Haenszel fixed-effect method to calculate the ORs for each APOE genotype and allele, using all other genotypes or alleles as the reference (Table 1). The heterogeneity of the included articles was evaluated using Cochran’s Q test and I^2^ statistics. *P* < 0.05 and I^2^ < 50% were considered statistically significant. When heterogeneity existed across the included studies (I^2^ < 50%), the random effect model was used^15^; when there was no heterogeneity across the included studies (I^2^ > 50%), the fixed-effect model was used (Table 1)^15^. Funnel plots were used to explore the publication bias for all ApoE alleles and genotypes^16^. Egger’s regression test and Begg’s rank test were used to assess asymmetry, and the Mathur and VanderWeele method was used supplementally to detect the impact on publication bias within each pairwise comparison (Fig. 4). To assess whether our results were substantially influenced by the presence of any individual study, we conducted a sensitivity analysis by systematically removing each study and recalculating the significance of the results. A subgroup analysis was performed to verify the reliability of the results in articles reporting sporadic AD (not described in other articles), and forest maps were drawn (Fig. 5).

**Table 1:** Meta-analysis of Apolipoprotein E gene polymorphism with AD in full model.

	n	I^2^	H	Q (P)	Fix-effect model/random-effect model	OR	OR (95% CI)
**Allele**							
ε2	116	55.8%	1.50	260.69*	Fix-effect model	0.7405*	0.7063–0.7764
					Random-effect model	0.7709*	0.7051–0.8428
ε3	116	68.30%	1.78	363.08*	Fix-effect model	0.5774*	0.5599–0.5954
					Random-effect model	0.5390*	0.5040–0.5764
ε4	116	69.4%	1.81	375.90*	Fix-effect model	2.5962*	2.5001–2.6959
					Random-effect model	2.8474*	2.6114–3.1014
**Genotype**							
ε2/ε2	66	0.00%	1.00	59.48 (0.6699)	Fix-effect model	0.6118*	0.5039–0.7429
					Random-effect model	0.6525*	0.5292–0.8044
ε2/ε3	116	45.4%	1.35	210.72*	Fix-effect model	0.6405*	0.6050–0.6782
					Random-effect model	0.6485*	0.5845–0.7144
ε2/ε4	109	36.7%	1.26	170.73*	Fix-effect model	1.4008*	1.2582–1.5497
					Random-effect model	1.5213*	1.2696–1.8229
ε3/ε3	116	66.7%	1.73	344.87*	Fix-effect model	0.5514*	0.5309–0.5725
					Random-effect model	0.5077*	0.4680–0.5507
ε3/ε4	116	60.1%	1.58	288.30*	Fix-effect model	2.2670*	2.1655–2.3734
					Random-effect model	2.4913*	2.2666–2.7383
ε4/ε4	106	16.7%	1.10	126.11 (0.0786)	Fix-effect model	5.4812*	4.8014–6.2573
					Random-effect model	4.9588*	4.0694–6.0427

*P < 0.001.

Statistical analyses were performed in R (version 4.0.5 for Windows) using the metafor (version 3.0-2) and meta (version 4.19-2) meta-analysis packages. A value of *P* < 0.05 was considered significant, and *P* values were two-sided. Because of the potential for type I error caused by multiple comparisons, findings for subgroup analyses and secondary outcomes were interpreted as being exploratory.

## Results

### Study characteristics

**Figure 1:**
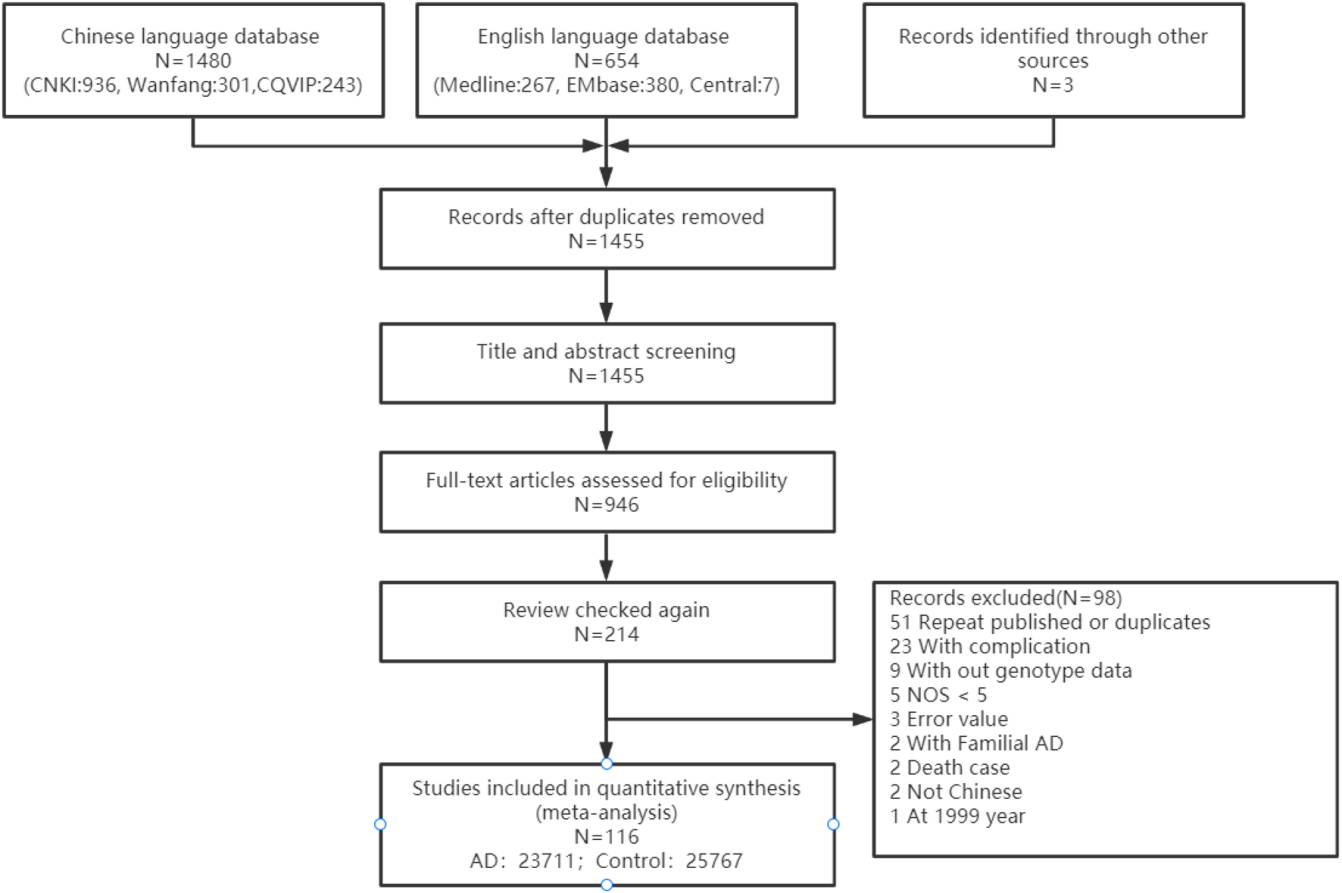
The study screening process.

**Figure 2:**
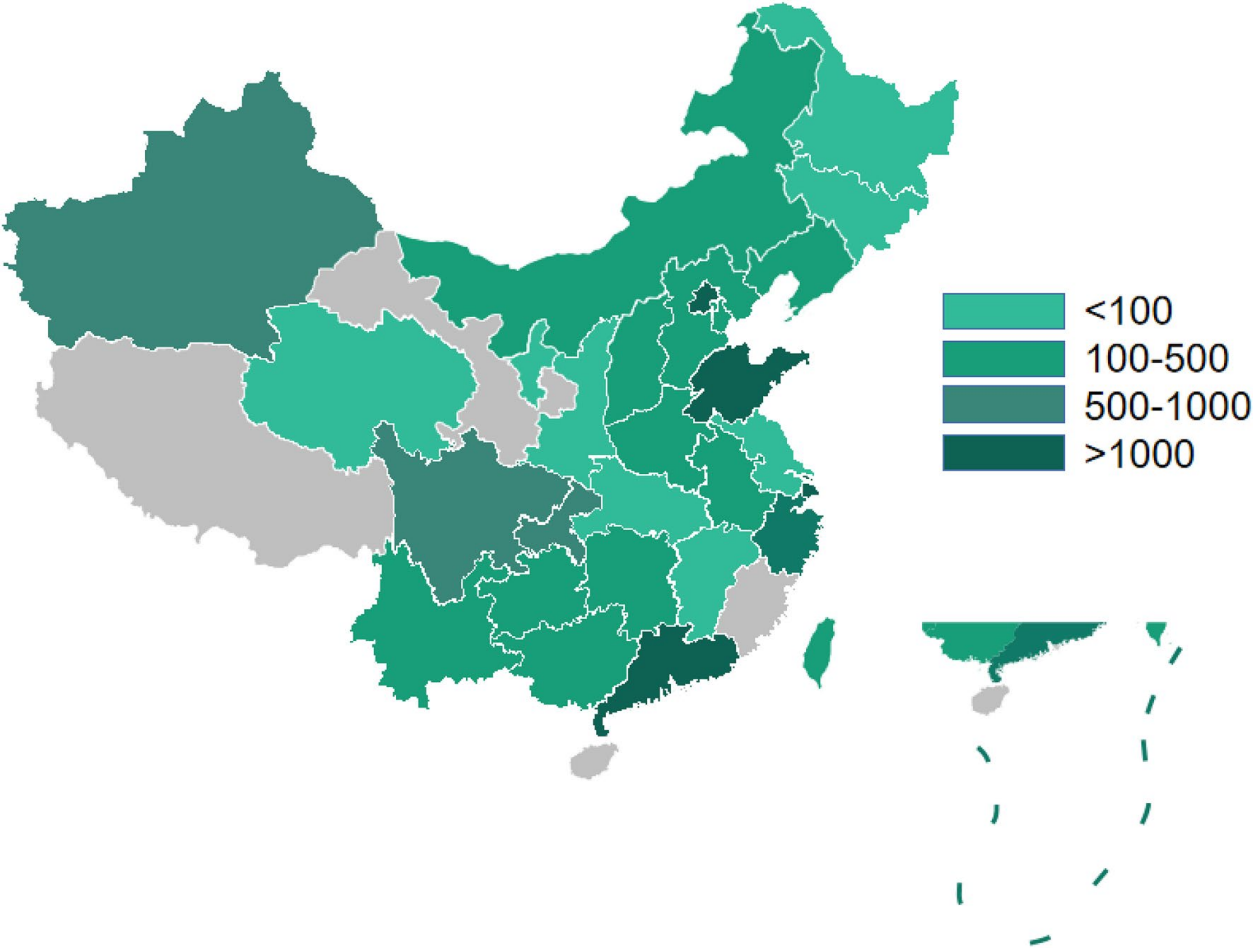
115 studies participants from various geographical areas in China.

**Figure 3:**
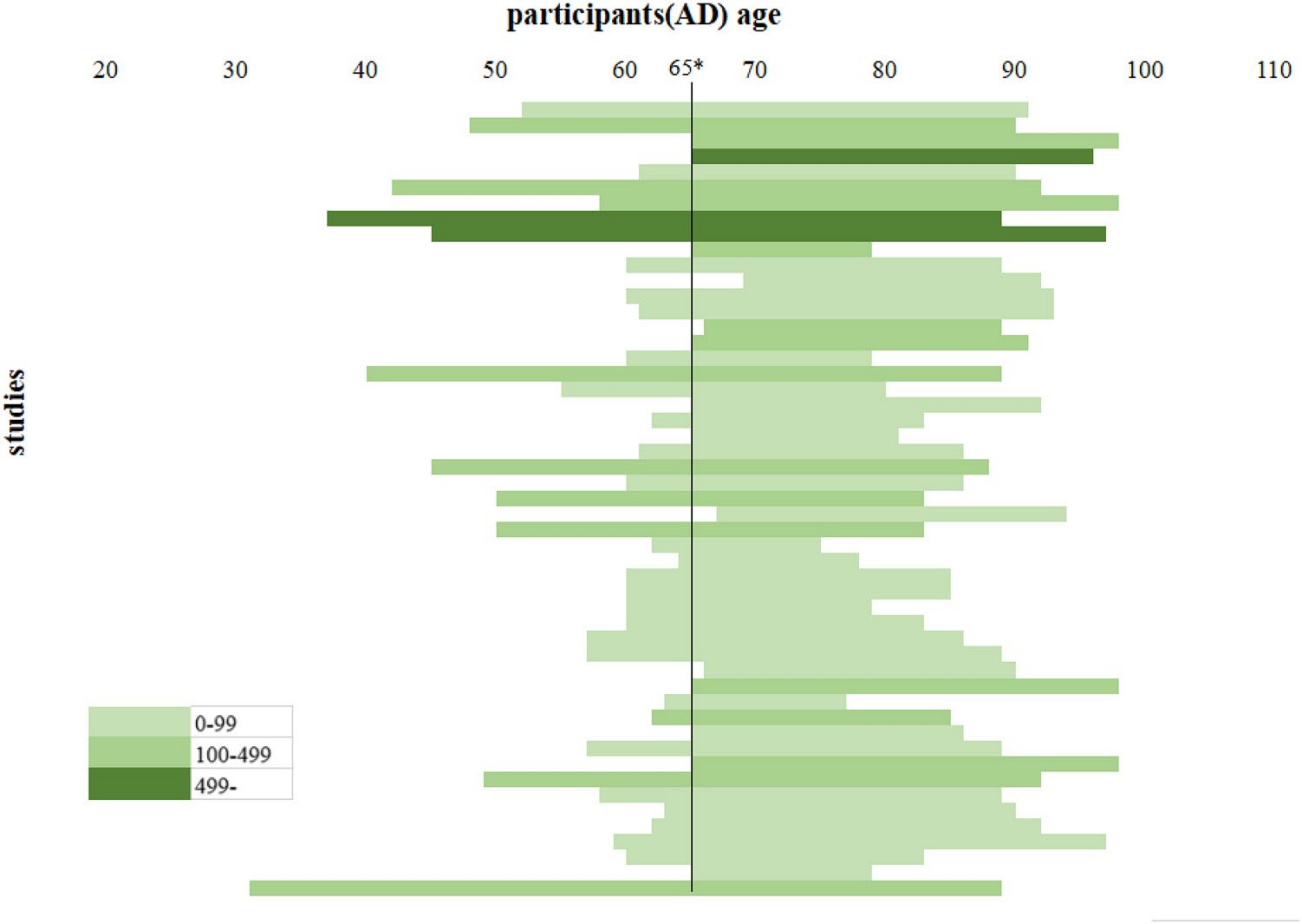
Age distribution of subjects in 51 studies. *65 is the dividing line between early-onset Alzheimer’s and late-onset Alzheimer’s.

Among the 2134 articles retrieved from the databases, 116 articles were finally screened: 96 Chinese articles (Supplementary) and 20 English articles^17–36^. The articles included a total of 23,396 patients with AD and 25,568 control individuals. Figure 1 depicts the literature screening process. The study area source distribution was as follows: a total of 115 studies clearly identified the specific source of cases covering 28 provincial-level regions (Fig. 2). Another multicentre study, which could not be consolidated, covered 30 provincial-level regions^21^. Fifty-one of the studies reported the age distribution of the subjects (Fig. 3). In conclusion, the case sources in this meta-analysis covered at least 30 provincial-level administrative regions (including Taiwan and China) out of 34, with good representation.

### Association between apolipoprotein E genotype and Alzheimer’s disease

Table 1 lists the results obtained for each allele and genotype of ApoE in this meta-analysis. With the exception of the ε2 homozygotes and ε4 homozygotes, all I^2^ > 50% and Q statistics were significant. Therefore, the corresponding random model and fixed model were used for calculating the pooled OR. ApoE ε2/ε4, ε3/ε4 and ε4/ε4 were significantly associated with AD (ε2/ε4: OR 1.521, 95% CI [1.270–1.823], *P* < 0.001; ε3/ε4: OR 2.491, 95% CI [2.267–2.738], *P* < 0.001 and ε4/ε4: OR 5.481, 95% CI [4.801–6.257], *P* < 0.001). The ε2/ε2, ε2/ε3 and ε3/ε3 genotypes were also significantly associated with AD (ε2/ε2: OR 0.612, 95% CI [0.504–0.743], *P* < 0.001; ε2/ε3: OR 0.649, 95% CI [0.585–0.714], *P* < 0.001 and ε3/ε3: OR 0.508, 95% CI [0.468–0.551], *P* < 0.001). The OR of ε4 homozygotes was the largest, and that of ε3 homozygotes was the smallest.

### Association between the apolipoprotein E allele and Alzheimer’s disease

Table 1 lists the results obtained for each allele and genotype of ApoE in this meta-analysis. The I^2^ > 50% and Q statistics were significant; thus, heterogeneity between studies was significant. The random effect model was applied for calculating the pooled OR. The frequency of the ApoE ε4 allele was higher in patients with AD than it was in healthy controls and exhibited a statistically significant positive association between risk factor ε4 allele carriers and AD in the Chinese population (OR 2.847, 95% CI [2.611–3.101], *P* < 0.001). The frequency of ApoE ε3 was lower in patients with AD than it was in healthy controls, and the difference was also statistically significant (OR 0.539, 95% CI [0.504–0.576], *P* < 0.001). This finding implies the protective effect of the ε3 allele regarding the development of AD in the Chinese population. The ε2 and ε3 alleles yielded the same result (OR 0.771, 95% CI [0.705–0.843], *P* < 0.001). However, the OR of ε2 was closer to 1 than that of ε3.

### Publication bias and sensitivity analysis

**Figure 4:**
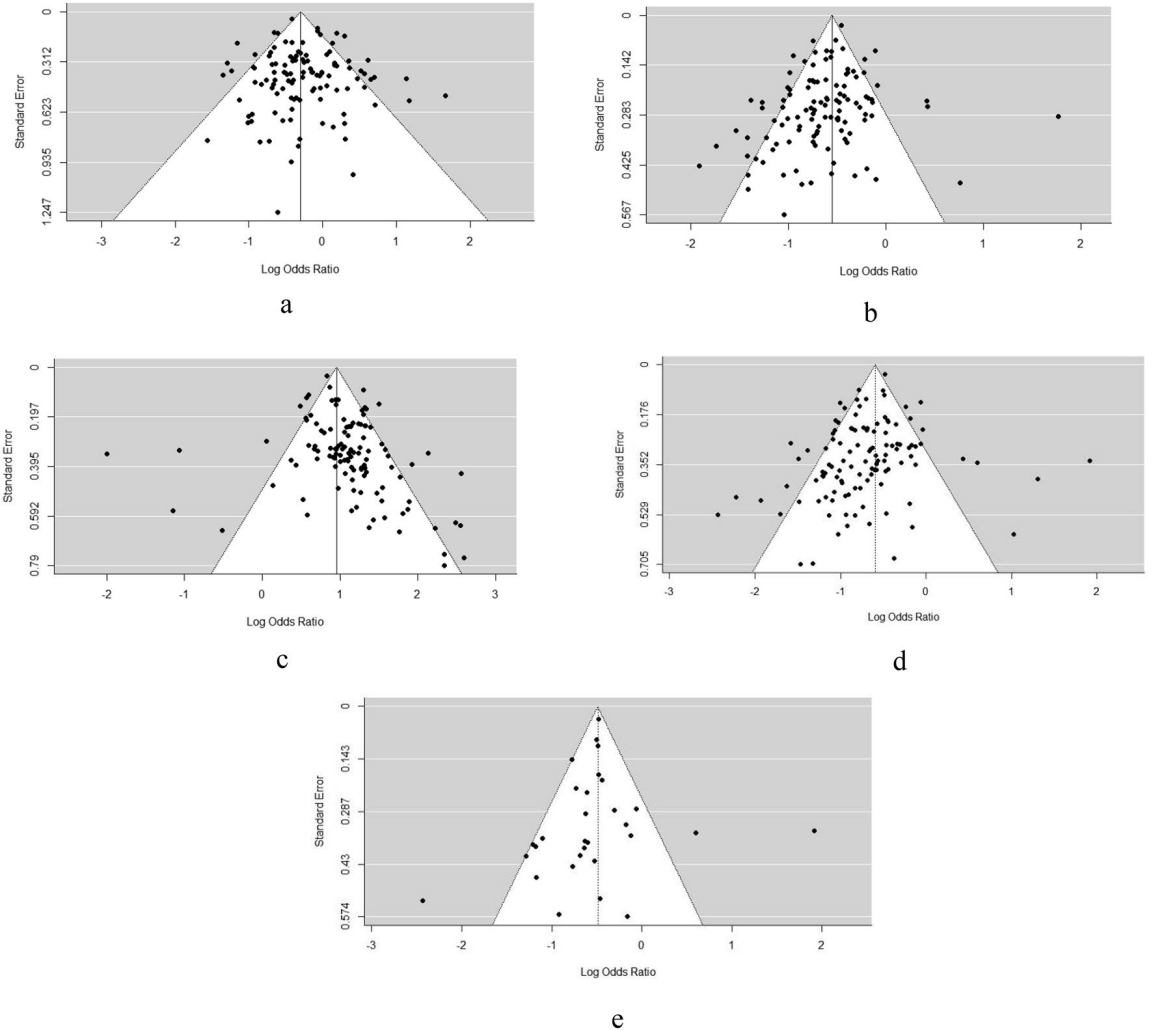
(**a**) Funnel plot on the association between ε2 Allele and AD in full model. (**b**) Funnel plot on the association between ε3 Allele and AD in full model. (**c**) Funnel plot on the association between ε4 Allele and AD in full model. (**d**) Funnel plot on the association between ε3/ε3 Genotype and AD in full model. (**e**) Funnel plot on the association between ε3/ε3 Genotype and AD in subgroup analysis model.

Figures 3 and 4 show the distribution of the ORs from individual studies in relation to their respective standard deviation in the funnel plot. There was a possibility of a publication bias risk in the meta-analysis. To eliminate any publication bias, the random effect model was selected for most alleles and genotypes. By systematically deleting each study each time and recalculating the results, the OR values were all close to the original results, with a small fluctuation range and significance. All sensitivity analyses were consistent with the primary results.

### Subgroup analysis

**Figure 5:**
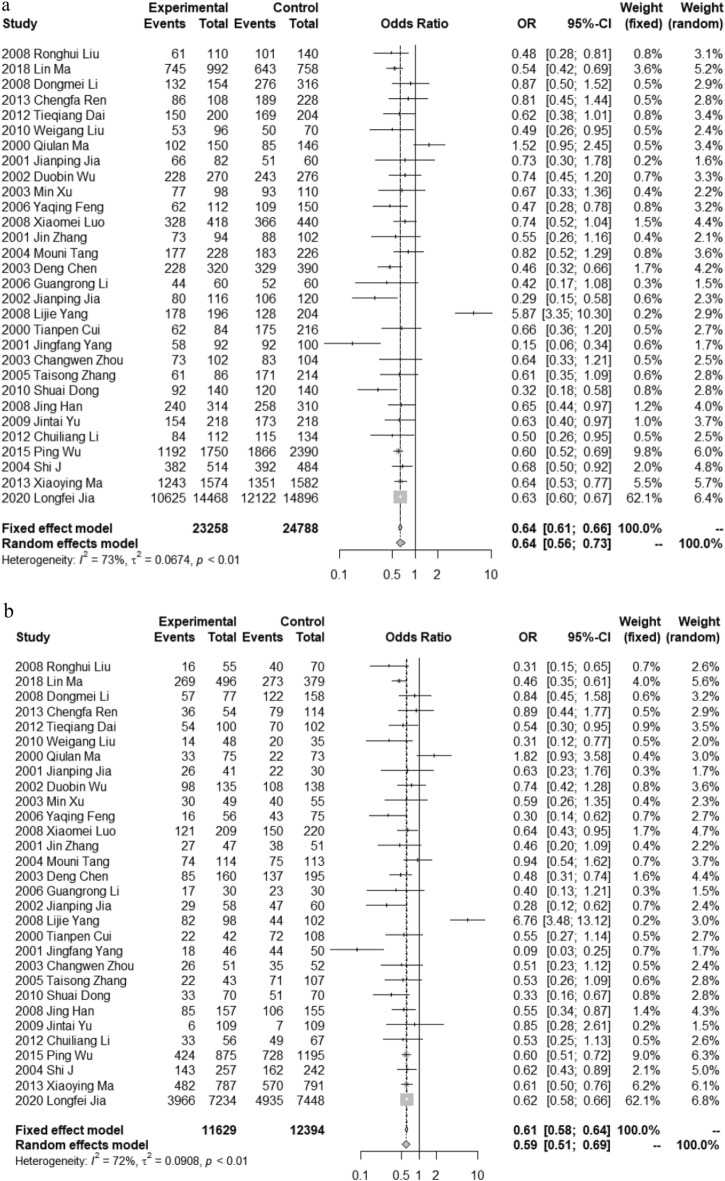
(**a**) Forest plot on the association between ε3 allele carriers and AD in subgroup analysis. (**b**) Forest plot on the association between ε3/ε3 Genotype carriers and AD in subgroup analysis.

As further verification of the ‘protective effect’ of the ε3 allele, funnel plot results showed high heterogeneity in all studies. A subgroup analysis was performed using the same method for the inclusion of data from the literature that explicitly stated that the study object was sporadic AD. Because of the different genetic relationships between APOE and sporadic AD and familial AD, sporadic AD was predominant in the majority of articles; therefore, studies that clearly stated that the study object was sporadic AD were included in the subgroup analysis. The screening results of 30 references (the sporadic/familial type was not described in the remainder of the articles) were as follows: AD: 11,629 cases, control: 12,394 cases; sex ratio: AD male/female: 5543/5476; control male/female: 5489/6383 (three studies did not provide the sex ratio), gender difference between the two groups, *χ*^2^ = 37.900, *P* < 0.001. Figure 5a,b report the subgroup analysis of ε3 and the ε3/ε3 forest diagram, respectively. The frequency of ApoE ε3 in sporadic AD was lower than that in healthy controls, and the differences were statistically significant (OR 0.642, 95% CI [0.564–0.731], *P* < 0.001): ε2/ε3 (OR 0.668, 95% CI [0.555–0.804], *P* < 0.001) and ε3/ε3 (OR 0.595, 95% CI [0.510–0.694], *P* < 0.001). The frequencies of alleles and genotypes were lower than those observed in the healthy controls, and the differences were statistically significant. The OR value of ε3/ε3 was smaller than that of ε2/ε3, which was consistent with the results of the whole model.

## Conclusion

These results provide support for the protective effect of the ApoE ε3/ε3 allele regarding the development of AD in the Chinese population.

## Discussion

For a long time, the ε4 allele of ApoE was considered the ‘risk’ variant for several phenotypes compared with the ε3 (‘neutral’) and ε2 (generally considered as ‘protective’, although less consistently) alleles^10^. Nevertheless, none of the studies that reached this conclusion used the Chinese population as the main research object, and there is some evidence that ε3 is not ‘neutral’ in the Chinese population or even in the East-Asian population. This research was the most comprehensive meta-analysis of the correlation between APOE status and AD in the Chinese population. Based on the distribution of the *APOE* gene in AD reported in the Chinese population in the last 20 years, it is concluded that the APOE ε3 allele has a protective effect on sporadic AD in the Chinese population and that its protective effect is stronger than that of ε2^10^. This is of great significance. In turn, it is not suggested that the APOE ε3/ε3 genotype be used as the reference in future studies of sporadic AD in the Chinese population. The limitation of this research lies in the fact that most of the studies included here were not clear about the familial AD and sporadic AD status. Because the genetic factors of familial AD are different from those of sporadic AD, a subgroup analysis was adopted to reduce this bias. The follow-up study of familial AD can be based on the case data of The Chinese Familial Alzheimer’s Disease Network. Based on the results of this study and the meta-analysis performed in another Chinese population^10^, which reported that ‘No significant association was found between ApoE ε2 alleles and AD’, we propose the hypothesis that the protective effect of APOE ε3 is stronger than that of ε2 in sporadic AD in the Chinese population, which remains to be proven in future studies.

In conclusion, our meta-analysis suggests that ApoE ε4 carriers have a higher risk of AD and provides support for the protective effect of the ApoE ε3 allele against the development of AD in the Chinese population. Although ApoE ε3 is not a well-studied genetic protection factor for the development of AD, in some regions, most non-AD subjects carry this genotype. This will provide important insights into the prevention of AD.

## Supplementary Information

Supplementary Information 1.

Supplementary Information 2.

## Data Availability

All data generated or analysed during this study are included in this published article [and its Supplementary Information files].
