# Supplementary material for: Protective effect of apolipoprotein E epsilon 3 on sporadic Alzheimer’s disease in the Chinese population: a meta-analysis
Source: Sci Rep. 2022 Aug 10;12:13620. doi: 10.1038/s41598-022-18033-x (PMC9365782; doi:10.1038/s41598-022-18033-x)
Supplement: Supplementary file 2 — Supplementary Information 2. [file 41598_2022_18033_MOESM2_ESM.docx]

**This is the supplementary document required by the Reviewer 2.**

**References(**supplementary)

[1] DUAN Guan-min, et al. Study on the Association of Apolipoprotein E gene polymorphism with Demented Patients[J]. Journal of Henan Medical College for Staff and Workers, 2009, 21(6): 559-561.

[2] Fan Ping, et al. Serum Apolipoprotein A, B100 and E Levels and Apolipoprotein E Polymorphism in Patients with Alzheimer's Disease and Multipleinfarction Dementia in Chinese Population[J]. Journal of West China University of Medical Sciences, 2001(3): 389-391.

[3] LIU Rong-Hui, et al. The relationship between Aβ40, Aβ42 levels and ApoE gene polymorphism of Alzheimer's disease patients[J]. Chinese Journal of Gerontology, 2008(13): 1292-1293.

[4] Lv Ze-ping, et al. APOE and LRP gene polymorphisms are associated with clinical symptoms in patients with dementia[J]. Chin J Neuroimmunol & Neurol, 2013, 20(5): 331-334.

[5] SHI Yu-ling. Retrospective analysis of ApoE gene polymorphism and Hcy level in 306 patients with senile dementia[J]. Chin J Lab Diagn, 2019, 23(6): 973-976.

[6] Rang Wei-hua. Association of ApoE、 TNF- α -850C/T Gene Polymorphisms with Alzheimer's Disease[D]. Guangzhou Medical University, 2011.

[7] Zhang Ting. APOE and MTHFR gene polymorphism analysis in the research of alzheimer disease's genetic susceptibility[D]. Southern Medical University, 2016.

[8] Ma Lin. Study on the role of ApoE, ADH1B rs1229984 and ALDH2 rs671 gene polymorphisms in patients with Alzheimer's disease of Han population in Inner Monglia[D].Wuhan University, 2018.

[9] Zhu Jiyng, et al. The association of apolipoprotein E and Alzheimer's disease[J]. Practical Geriatrics, 2000(1): 23-25.

[10] Gao Yanxia, et al. Correlation analysis of ApoE, CYP46 gene polymorphism and Alzheimer's disease[J]. CHINESE JOURNAL OF INTEGRATIVE MEDICINE ON CARDIO-/CEREBROVASCULAR DISEASE, 2009, 7(3): 314-315.

[11] Dai Tie-qiang. The relationship between polymorphism of ApoE gene and cognitive function in SAD[D]. Central South University, 2012.

[12] Ren Chen-fa. The Study on the asssociation of Apolipoprotein E Gene Polymorphism with Alzheimer's disease and Mild Cognitive Impairment among han elderly people in Jiangxi[D]. Nanchang University, 2013.

[13] Chen Long. The association Between the Common Variations of ApoE and TRIP4 Genes and Alzheimer disease[D]. Anhui Medical University, 2016.

[14] Xiao Xiao.Relationship between ApoE Gene polymorphism and Behavioral of Frontotemporal Dementia[D]. Zhengzhou University, 2019.

[15] GU Zhi-lei, et al. Measurement of Polymorphism of Apolipoprotein E Genotype and Its Correlation to Alzheimer's Disease[J]. Acta Universitatis Medicinalis Secondae Shanghai, 2001(4): 325-327.

[16] LI Dong-mei, et al. Association of Apolipoprotein E Polymorphism and Sporadic Alzheimer's Disease[J]. J Mod Lab Med, 2008(2): 20-23.

[17] Lu Shen-ji. The association of LRRK2 Variants with Alzheimer's disease of Han Chinese Population[D]. Fudan University, 2012.

[18] Sun Yan. Konck-down the expression of human ZNF313 gene by RNAi technology and The association study of genetic polymorphisms and Alzheimer's disease[D]. Sichuan University, 2005.

[19] YANG Jia-yi, et al. Study of the polymorphisms of APOE gene in alzheimer disease and vascular dementia[J]. Nervous Diseases and Mental Health, 2008(2): 138-140.

[20] Jiang Sanduo, et al. Association analysis of expression of Aβ with APOE gene and PS1 gene in Alzheimer's disease[J]. Shanghai Archives of Psychiatry, 2000(1): 13-16.

[21] FENG Ya-qing, et al. Analysis of the polymorphisms of apoE gene and ACE gene in Alzheimer's disease and vascular dementia[J]. Chin J Geriatr Heart Brain Vessd Dis, 2004(3): 181-183.

[22] Cui Jingbin, et al. Effect of apolipoprotein E genotype on plasma apolipoprotein E, total cholesterol and triglyceride levels in patients with Alzheimer's disease[J]. Shanghai Med J, 2002(7): 444-446.

[23] FU Xueting, et al. Study on the relationship between the syndrome types of Alzheimer'sdisease in Uyghur medicine and ApoE gene polymorphism[J]. Journal of Xinjiang Medical University, 2016, 39(11): 1471-1473, 1477.

[24] DONG Xiang. The Correlation Analysis of AD Patients’ Mental and Behavioral Disorders and ApoE and LRP Gene Polymorphism[J]. Guide of China Medicine, 2013, 11(18): 455-456.

[25] LIU Wei-Gang, et al. Relation between the levels of glucose and true insulin in cerebrospinal fluid and apolipoprotein E genotype in patients with Alzheimer′s disease[J]. Practical Geriatrics, 2010, 30(3): 291-293.

[26] Du Hong-jian. Study on Apolipoprotein E gene polymorphism and neuroelectrophysiology in Alzheimer's disease[D]. Tianjin Medical University, 2002.

[27] Wu Li, et al . A Study on the Relationship between Polymorphisms of APOE Gene and Psychological Symptoms in Alzheimer Disease[J]. China Journal of Health Psychology, 2008(10): 1168-1170.

[28] CAI Haiyan, et al. Correlation between apolipoprotein E gene and Alzheimer's disease[J]. Ningxia Med J, 2013, 35(12): 1149-1150.

[29] LI Jie, et al. The analysis of apolipoprotein E geng genotype of patients with Alzheimer's Disease[J]. Journal of Tianjin Medical University, 2001(1): 6-8.

[30] LUO Xiao-mei, ZHOU Xiao-hui. The study of Apolipoprotein E gene polymorphism with Alzheimer's Disease[J]. Journal of Xinjiang Medical University, 2008, 148(4): 388-390.

[31] JIA Jian-ping, et al. A study on ApoE genotype in Alzheimer-type dementia and vascular dementia[J]. J Apoplexy and Nervous Disease, 2001(2): 3-5.

[32] LONG Yan, et al . Influences of EBV infection and single nucleotide polymorphism of apolipoprotein E gene on Alzheimer's disease[J]. Chin J Public Health, 2008(5): 573-574.

[33] MA Qiulan, et al. Analysis of the interaction of the polymorphisms of presenilin 1 gene and ApoE gene in Alzheimer's disease[J]. Chin J Med Genet, 2000(6): 17-21.

[34] Xing Tao-tao. Blood Clusterin Levels, rs9331888 Polymorphism and the Risk of Alzheimer's Disease[D]. Qingdao University, 2012.

[35] Yu Nan-nan. Tau-tubulin kinase-1 polymorphisms and risk of late-onset Alzheimer's disease[D]. Qingdao University, 2016.

[36] Wu Duo-bin. Analysis of ps-1, ApoE, and MTHFR polymorphisms or mutations in patients with dementia[D]. PLA Military Medical Training College, 2002.

[37] LAI Shilong, et al. Association between apolipoprotein E polymorphism and Alzheimer's disease: A population-based study in Guangzhou, China[J]. Chin J Epidemiol, 2001(3): 46-48.

[38] BI Sheng, et al. Two polymorphisms in the LRP gene and Alzheimer's Disease[J]. Chin J Neurol, 2000(1): 13-16.

[39] Yu Min, Jia Jian-ping. Estrogen receptor α and apolipoprotein E gene polymorphisms and sporadic Alzheimer's disease[J]. Natl Med J China, 2003(24): 70-72.

[40] MAI Yi-cheng, et al. Association between Interleukin-8 and Apolipoprotein E Gene Polymorphism and Late-onset Alzheimer's Disease[J]. JOURNAL OF SUN YAT-SEN UNIVERSITY(MEDICAL SCIENCES), 2010, 31(1): 118-121.

[41] Shang Yin. The risk factors of Alzheimer's disease among Tibetan aged 60 years and older in Qinghai Province [D]. Southern Medical University, 2015.

[42] Zhou Lan. Preliminary study on the characteristics of TCM syndrome differentiation and APOE gene polymorphism in senile dementia[D]. Hubei University Of Traditional Chinese Medicine, 2004.

[43] Qian Hai-rong. Prion Protein Gene and cellular Prion Protein: Correlations with Aging and Alzheimer's Disease[D]. PLA Military Medical Training College, 2007.

[44] Ma Ai-jun. The study on the relationship between Parkinson disease with dementia and Alzheimer disease[D]. Tianjin Medical University, 2007.

[45] LIU Bo, MENG Fanchao. The relationship between serum apolipoprotein E gene polymorphism and amyloid β protein precursor 16-17 exogenous factor expression in patients with Alzheimer's disease and its clinical significance[J]. Chinese Journal of Practical Nervous Disease, 2020, 23(3): 185-189.

[46] CHENG Liu, et al. A Study of Association between Apolipoprotein Eε4 Allele and Senile Dementia[J]. Chin J Environ Occup Med, 2002(4): 213-215.

[47] YUAN Yong-gui, et al. A Study of Serum Lipid Concentrations and Apolipoprotein E Genotype among Patients with Senile Depression and Alzheimer Disease[J]. Chinese General Practice, 2006(2): 106-108.

[48] ZHANG WEI,TAN LAN, JIANG SAN DUO. A CORRELATION BETWEEN APOLIPOPROTEIN E POLYMORPHISM AND β AMYLOID PRECURSOR PROTEIN IN PATIENTS WITH ALZHEIMER DISEASE[J]. Med J Qilu, 2006(4): 293-295, 297.

[49] Lv Xiaorong, Zhong Yuan. Association of apolipoprotein E gene polymorphism with mild cognitive impairment and Alzheimer's disease[J]. Chinese Journal of Gerontology, 2012, 32(5): 917-919.

[50] Zhang Yan. Study on the relationship between TCM Syndrome Types and ApoE gene polymorphism in Xinjiang Kazak[D]. Xinjiang Medical University, 2017.

[51] LUO Xiao-mei. Xinjiang Uygurs and Hans Apolipoprotein E gene polymorphism analysis of Alzheimer's Disease[D]. Xinjiang Medical University, 2008.

[52] Zhu Weiming, et al. Relationship between mental behavior disorder and ApoE, LRP, α 2-Mg gene polymorphisms in Alzheimer's disease patients in Wenzhou[J]. Fujian Med J, 2011, 33(5): 87-89.

[53] FENG Ya-qing, et al. Correlation of the polymorphisms of apolipoprotein E gene and low-density lipoprotein receptor-related protein gene with sporadic Alzheimer’s disease[J]. Journal of International Neurology and Neurosurgery , 2006(1): 9-12.

[54] LU Zheng, et al. An analysis of association between apolipoprotein E gene and Alzheimer disease in a community population of Shanghai[J]. Chin J Psychiatry, 2001(1): 18-21.

[55] Ma Cui, et al. Genetic association among polymorphism in presenilin 1 gene, ApoE gene and late-onset Alzheimer's disease[J]. Chin J Nerv Ment Dis, 2000(6): 345-347.

[56] Zhang Wei. The association Among Apolipoprotein E Polymorphism β amyloid protein and Vascular Dementia[D]. Qingdao University, 2004.

[57] Zhang Ai-ping. Relationship of Hcy and its related enzyme's gene polymorphisms with Alzheimer's disease[D]. Shanxi Medical University, 2007.

[58] Wang Yu. The study on the relationship between inflammatory factors and Parkinson disease and Alzheimer disease[D]. Tianjin Medical University, 2010.

[59] ZHANG Jin, et al. An analysis of apolipoprotein E polymorphism in patients with vascular dementia[J]. Chin J Geriatr Heart Brain Vessd Dis, 2001(1): 24-27.

[60] Pei Wenyi, Jiang Haoming, Xie Li. Relationship between serum apolipoprotein E, Hcy levels and the disease severity, types of dementia patients[J]. Chin J Clin Health, 2020, 23(1): 65-69.

[61] Zhang Yong, et al. The relationship between polimorphisms in apolipoprotein E gene and vascular dementia or Alzheimer's diseases[J]. Shandong medicine Journal, 2004(22): 1-2.

[62] CHEN Yun, et al. Genetic relations of the LDL receptor-related protein gene and apolipoprotein E gene to Alzheimer disease[J]. J Shanxi Med Univ, 2006(6): 565-568.

[63] ZENG Xiangyu, et al. Effects of apolipoprotein E gene on the metabolism of serum lipids in patients with Alzheimer’ s disease[J]. Chin J Psychiatry, 2002(2): 26-29.

[64] TANG Mu-ni, et al. Analysis on association between the polymorphisms in apolipoprotein E, interleukin-1α genes and Alzheimer's disease in Chengdu area[J]. Chin J Med Genet, 2004(2): 84-86.

[65] Xie Xin-xin. Correlation between Apolipoprotein E Gene Polymorphism and the Urine Level AD7c-NTP in Patients with Alzheimer's Disease and Mild Cognitive Impairment[D]. Anhui Medical University, 2018.

[66] CAO Li-chun, et al. Correlation Between Apolipoprotein E Gene Polymorphism and Alzheimer and Coronary Heart Disease[J]. Journal of Chinese Physician, 2003(6): 741-743.

[67] LI Guangrong, et al. Polymorphism in the Apolipoprotein E Gene and Its Association with Sporadic Alzheimer Disease[J]. JOURNAL OF GUIYANG MEDICAL COLLEGE, 2006(1): 24-26, 29.

[68] CHEN Deng, et al. Apolipoprotein E Gene Polymorphisms and Alzheimer Disease[J]. Acta Genetica Sinica, 2003(12): 1167-1170.

[69] DONG Xiang. Relationship between apolipoprotein E gene polymorphism and Alzheimer's disease and Parkinson's disease dementia[J]. Guide of China Medicine, 2014, 12(24): 206-207.

[70] Cui Tianpeng, et al. Relationship between apolipoprotein E gene polymorphism and sporadic Alzheimer's disease[J]. Chinese Journal of Pathophysiology, 2000(8): 70-72.

[71] Yang Lijie. Association between apolipoprotein E gene polymorphism and sporadic Alzheimer's disease[J]. CHINA MODERN DOCTOR, 2008, 46(24): 89-90.

[72] JIA Jian-ping, et al. Association between the apolipoprotein E gene polymorphism and the genetic sensitivity of sporadic Alzheimer's diseases[J]. Chin J Geriatr, 2002(4): 7-9.

[73] Wang Jin-tao. The Association study of Apolipoprotein E Gene Polymorphism with Different Types of Dementia in a Chinese Han Population[D]. Peking Union Medical College, 2010.

[74] Yang Jingfang, et al. Association between ApoE gene polymorphism and Alzheimer’ s disease and Carotic arteriosclerosis in Chinese[J]. 基础医学与临床, 2001(1): 40-42.

[75] Wu Ping, et al. Relationship Between Apolipoprotein E Gene Polymorphism and Early-onset Alzheimer’ s Disease[J]. Chin J Clin Neurosci, 2009, 17(3): 261-266.

[76] Wang Qingfeng. The significance of apolipoprotein E genotyping in the diagnosis of dementia[J]. Practical Geriatrics, 2003(4): 209-210.

[77] ZHOU Chang-wen, et al. Genetic correlation study between apolipoprotein E gene and Alzheimer's disease[J]. Med J NDFNC, 2003(4): 244-246.

[78] Xiao-Ping Zhao, et al. Relationship of apolipoprotein E gene polymorphism to Parkinson's disease and Alzheimer's disease[J]. Chinese Journal of Clinical Rehabilitation , 2003(31): 4262-4263.

[79] Mou Shan-mao. Association between chromosome 9p21.3 rs1333049 single nucleotide polymorphism and Late-onset Alzheimer disease in Chinese Han population[D]. Taishan Medical College, 2011.

[80] Liu Xiao-yan. Association analysis of late-onset Alzheimer's disease and susceptibility genes in Chinese Han population[D]. Central South University, 2014.

[81] Zhou Yong-tao. Association of Family History and polymorphism in ACT-51G/T, IL-1α-889C/T, LRP766C/T and NOS-Ⅲ298G/T with Alzheimer’s Disease in Chinese Han Population[D]. Peking Union Medical College, 2005.

[82] HUANG Wen, et al. The frequency distribution of apolipoprotein E genotypes in elderly people in Chongqing area and its relationship with senile dementia onset[J]. ACTA ACADEMIAE MEDICINAE MILITARIS TERTIAE, 2003(2): 160-163.

[83] ZHOU Chang-long, et al. Relationship between Apolipoprotein E Polymorphism and Alzheimer’ s Disease in Chongqing[J]. Chinese General Practice, 2012, 15(27): 3115-3117.

[84] Sheng Bi, et al. Association of interleukin I beta and apolipoprotein E polymorphism with Alzheimer's diseases in Chinese Han population[J]. Chinese Journal of Clinical Rehabilitation, 2004(16): 3068-3069.

[85] Yu Jin-tai. The Association study of ApoE and β2-AR Polymorphisms with Alzheimer's Disease in a Chinese Han Population[D]. Qingdao University, 2009.

[86] Zhang Tai-song. A study on the relationship between APOE and D10S1225 Polymorphisms and Alzheimer's Disease[D].Sun Yat-sen University, 2005.

[87] Wang Hui-fu. SORCS1 and APOE polymorphisms interact to confer risk for late-onset Alzheimer's disease in a Northern Han Chinese population[D]. Qingdao University, 2013.

[88] PANG Guofang, et al. Study of cardiovascular risk factors in alzheimer dementia and vascular dementia[J]. Chinese Journal of Geriatric Care, 2015, 13(4): 28-29, 33.

[89] Han Jing. Analysis of the levels of serum lipids in patients with Sporadic Alzheimer's Disease in xinjiang and related study of ApoE Polymorphism[D]. Xinjiang Medical University, 2008.

[90] Wu Ping. Frequencies of ApoE Gene Polymorphism and PPC Allele Length Polymorphism at the Upstream Region of cav-1 Gene and Association of these Polymorphisms with AD, MCI and VD[D]. Fudan University. 2009.

[91] Zhang Chen-hui. Status of ApoE Gene and SOM in Dementia Diagnosis, Analysis and Rehablication of BPSD[D]. First Military Medical University, 2006.

[92] DONG Xiang. The Study on the asssociation between AD disease and the ApoE gene Polymorphism, 2013, 23(4): 523-524.

[93] Wang Lei, et al. Study on sex hormone level and ApoE genotyping in Alzheimer's disease[J]. Journal of Beijing Normal University(Natural Science), 2008(3): 302-305.

[94] Xiao Ming-yue. Association of the polymorphisms of OGG1, ApoE gene with Alzheimer’ s disease[D]. Zhengzhou University, 2013.

[95] Li Chui-liang. An Association analysis of Apolipoprotein E Genotypes with Alzheimer's disease[D]. China Medical University, 2012.

[96] Dong Shuai. Association analysis between Alzheimer's disease and the intron short tandem repeat polymorphisms of Tau gene[D]. Guiyang Medical College, 2010.
